# A Preliminary Description of Companion Cat, Managed Stray Cat, and Unmanaged Stray Cat Welfare in Auckland, New Zealand Using a 5-Component Assessment Scale

**DOI:** 10.3389/fvets.2019.00040

**Published:** 2019-02-21

**Authors:** Sarah Zito, Jessica Walker, M. Carolyn Gates, Arnja Dale

**Affiliations:** ^1^Animal Welfare Science and Education Department, Royal New Zealand Society for the Prevention of Animal Cruelty, Auckland, New Zealand; ^2^New Zealand Companion Animal Council, Auckland, New Zealand; ^3^School of Veterinary Sciences, Massey University, Palmerston North, New Zealand

**Keywords:** cat management, unwanted cats, shelter medicine, stray cats, semi-owned cats, animal welfare, colony cats, cat welfare

## Abstract

Free-roaming cats are a polarizing issue in New Zealand and there is strong need for a comprehensive evaluation of their welfare to better inform population management decisions. In this study, a 5-component visual health-related welfare assessment scale was developed and piloted on a convenience sample of 213 free-roaming companion cats (CC), 210 managed stray cats (MS), and 253 unmanaged stray cats (UMS) from various locations in Auckland, New Zealand. The welfare assessment was performed through distance observation and consisted of body condition score (BCS); coat condition score; nose and eye discharge score; ear crusting score; and injury score. The majority of cats in all groups appeared generally healthy with no nose or eye discharge, ear crusting, or injuries. Although there were no appreciable differences in the apparent welfare of CC and MS cats, future studies with more robust sampling designs are needed to draw accurate inferences. The scale also requires further validation by comparing the visual observations against more detailed physical examination and biochemical data. Nonetheless, the results from this study provide preliminary information about assessing the health and welfare of stray cats as well as considerations for developing and implementing robust assessment scales.

## Introduction

In New Zealand, the Code of Welfare: Companion Cats (1) defines cats as belonging to one of three categories:

- Companion cats live with humans as “companions” and are dependent on humans for their welfare.
- Stray cats are companion cats who are lost or abandoned and who are living individually or in a group (colony). Stray cats have many of their needs indirectly supplied by humans, and live around centers of human habitation. Stray cats are likely to interbreed with the unneutered companion cat population.
- Feral cats are not stray cats and have none of their needs provided by humans. Generally, feral cats do not live around centers of human habitation. Feral cat population size fluctuates largely independently of humans, is self-sustaining, and is not dependent on input from the companion cat population.

New Zealand has one of the highest rates of cat ownership in the world with almost half of all households (44%) having at least one cat. There is an estimated total companion cat population of 1,134,000 in New Zealand and the majority of owned cats are at least partly free-roaming (2). There are also considerable numbers of stray cats in New Zealand; estimates indicate that there are approximately 196,000 stray cats in New Zealand, although the stray cat population is not able to be accurately quantified (3). Recently it has been suggested that the stray cat category should be further defined into managed and unmanaged stray cat categories. Managed stray cats have a human carer(s) who provides some care to the cat (feeding and sometimes other care such as veterinary care); unmanaged stray cats do not have a human carer(s) (4). The managed stray cat category includes, but is not limited to, cats referred to as colony cats (these are managed stray cats living within a specific cat colony) and semi-owned cats (these managed stray cats are of varying sociability, many are socialized to humans, they interact with people regularly and are directly and indirectly dependent on specific humans but are not part of a cat colony) (4, 5).

Concerns have been raised about the welfare state of stray cats, particularly when compared with companion cats (6–9). It has also been suggested that stray cats without carers suffer poorer welfare than cats in managed colonies who receive ongoing care from humans (10). Common welfare concerns include exposure of the cats to infectious diseases, the potential for cats to be injured or treated cruelly, and lack of adequate food and water resources (4, 7, 9, 11–15).

Concerns about cat welfare influence ethical cat management decisions and it is important that the choice of cat management strategy has no or minimal negative impact on cat welfare. The benefits to themselves reported by cat carers and their desire to continue to care for cats are sometimes used as justification for maintaining cat colonies (5, 16–18). However, the welfare of the cats should always be considered and given appropriate weighting vs. the needs and desires of cat carers.

In order to inform ethical cat management decisions, information about the welfare states of stray cats is vital in assessing whether the use of non-lethal return to field methods of cat management such as trap-neuter-return (TNR; where cats are sterilized and returned to live in their previous location), is appropriate in terms of cat welfare. If stray cats are known to generally suffer from poor welfare, then return to field cat management methods may not be ethically appropriate. However, if stray cat welfare is generally good then return to field cat management options should not be dismissed on cat welfare grounds.

To the authors' knowledge, a systematic welfare assessment of stray cats (managed stray cats with human carers and unmanaged stray cats without human carers) and companion cats has not been undertaken. The aim of this research was to collect empirical data on the welfare states of companion, managed stray, and unmanaged stray cats, piloting a new 5-compononent visual health-related welfare assessment as a tool to help inform ethical cat management decision making.

## Materials and Methods

### Development and Validation of Welfare Assessment Protocol

A 5-component visual health-related welfare assessment tool was developed in consultation with two veterinarians, one veterinary nurse, and two animal behaviorists. The assessment consisted of body condition score (BCS: emaciated, thin, ideal, overweight, over-condition or unknown/not recorded), which gives some information about the cats' nutritional and health status; coat condition score (poor, fair, good, excellent or unknown/not recorded), which gives some indication of the cats' general health status; nose and eye discharge score (none, mild, moderate, severe or unknown/not recorded), which can give some indications about whether the cats' are suffering from infectious disease such as feline upper respiratory tract infection; ear crusting score (none, mild, moderate, severe or unknown/not recorded), which can give some indications about whether the cats' are suffering from health problems such as ear mites or sun damage; and injury score (none, mild, moderate, severe or unknown/not recorded), which can give information about whether the cats have suffered an accident or injury. Only observable indicators of welfare were included as no direct contact with the cats could occur due to the welfare compromise that would have been inherent in handling the unmanaged stray cats.

**Figure 1:**
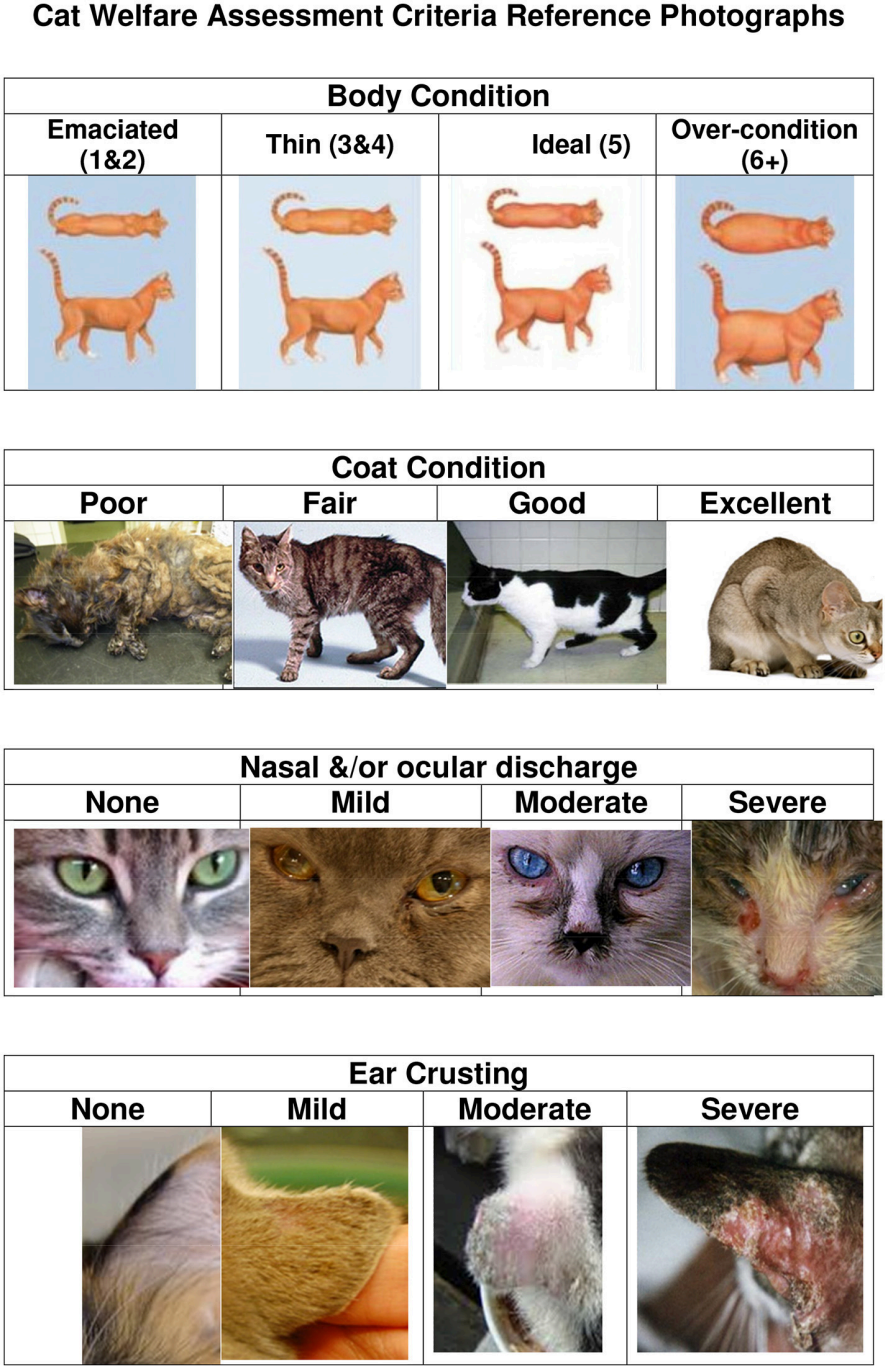
Cat welfare assessment criteria reference photographs.

The assessment was initially tested on a colony of approximately 100 cats and refined to maximize consistency between raters. No formal statistical testing of inter-and intra-rater reliability was conducted at the time the assessment was developed. However, an informal intra-observer reliability calibration was performed during the testing on the colony of cats used to test the assessment tool. This was done by discussing each cat with all researchers till agreement was reached. This was not repeated but two researchers assessed all cats except the companion cats and for consistency there was a calibration photo sheet (Figure 1) for all researchers to refer to for the different assessment items.

### Identification and Enrollment of Subjects

Cats were divided into companion, managed stray and unmanaged stray cats based on the definitions in the New Zealand Code of Welfare: Companion Cats (1). Companion cats (CC) were recruited for the study through friends, neighbors, veterinary clinics, and family of students who were involved in data collection. Advertisements were also placed in school newsletters and the local paper. Managed stray cats (MS) and unmanaged stray cats (UMS) were recruited via cat welfare organizations that work with managed and unmanaged stray cats in Auckland.

### Assessment of Cats

The welfare assessments were conducted by a team of 10 researchers over a 12-month period from November 2013 to November 2014 on a convenience sample of 676 cats from various unspecified locations in Auckland, New Zealand. The assessments for CC took place at the cats' homes with the owner present, while the assessments for MS cats were conducted when the animals were being fed by their carers; this allowed the researchers to be within a few meters of the cats to be able to observe them and carry out the welfare scoring. The assessments for UMS cats were conducted when the animals were trapped by the cat welfare organizations for other reasons; researchers were able to visually observe and welfare score the cats in the traps, or when the cats were removed from the traps at a shelter or veterinary clinic. The distance from which the cats were observed ranged from 1 to 5 m. In the case of colony cats, if the cats could clearly be seen they were scored, researchers were instructed that if it was not possible to see the whole cat or the cat was more than 5 m away the cat was not to be scored. However, it was possible to get within 5 m of all of the cats, including the unmanaged strays as these cats were all in traps and so were easily able to be assessed.

Cat demographic variables including color, coat length (categorized into short hair, medium hair, long hair, or unknown/not recorded), approximate age (categorized into juvenile, adult, or unknown), sex (categorized into male, female, or unknown/not recorded), and whether the cat was ear tipped (yes, no, or unknown/not recorded) were also recorded through visual observations. Companion cats' ages were recorded in years as indicated by the owners; this age was then used to categorize the cats into juveniles (<12 months of age) or adult (12 months of age or more). The carers of the managed stray cats provided an estimate of the age of the cat, based on whether the cat had joined the colony as a kitten or adult and how long the cat had been in the colony. The same age categorization as for companion cats was then applied. The unmanaged stray cats' age categorization was based on the information collected by the staff of the welfare organization, shelter or veterinary clinic when the cats were trapped as described previously. Categorization of the sex of the cats was based on information from the owners for companion cats, the cat carers and visual assessment for managed stray cats (whether the cats were ear-tipped), and visual assessment and information from the staff of the welfare organization, shelter or veterinary clinics for the unmanaged stray cats. Where sex could not be determined visually, the cat was recorded as being of unknown sex. It is common for free roaming stray cats to be ear-tipped when they are sterilized (19–24); this identifies the cat as a managed stray cat and should prevent a repeat surgery in error if the cat is re-trapped. Therefore, ear-tipping and information from the cats' carers were used to crudely estimate the percentage of MS and UMS who were sterilized since it was not possible to accurately assess the sterilization status through visual observation. Owners of CC were directly asked if their cat(s) had previously been sterilized.

Cats were visually assessed for the welfare assessment using a scoring sheet and the following health-based welfare assessments scored: body condition score (BCS; on a 9 point scale with 1–2 indicating emaciated, 3–4 thin, 5 ideal, and 6 or more over-condition), coat condition score (on a 4 point scale of poor, fair, good, and excellent), nose and eye discharge score (on a 4 point scale of none, mild, moderate, and severe), ear crusting score (on a 4 point scale of none, mild, moderate, and severe), and injury score (on a 4 point scale of none, mild, moderate, and severe; some specific injuries were recorded under comments). There was a calibration photo sheet for all researchers to refer to for the different assessment items (Figure 1). Animal ethics approval was not required as this was a purely observational study. Approval from the Unitec Human Ethics Committee was obtained for gaining informed consent from owners to participate in collecting data about their cats.

### Statistical Analysis

All data were imported into the R statistical software package for analysis (25). Descriptive statistics on the body condition score, coat condition score, nose and eye discharge score, ear crusting score, and injury score were provided for each of the different cat groups (CC, MS, and UMS cats). A statistical comparison between groups was not performed because of the known biases in the sampling methods and the inability to account for the random effects when multiple cats from the same colony or household were sampled.

## Results

The cats included in the study were 213 CC, 210 MS cats, and 253 UMS cats. Descriptive statistics on the demographic characteristics of the cats are reported in Table 1. Most cats were short haired (*n* = 535; 79%) and adults (*n* = 557; 82%). The majority of CC were reported by their owners as being sterilized (*n* = 195; 92%). Only 71 MS cats (34%) were ear-tipped and none of the UMS stray were ear-tipped.

**Table 1:** Demographic characteristics of 213 companion cats, 210 managed stray cats, and 253 unmanaged stray cats from Auckland, New Zealand.

		**Companion Cats (*n* = 213)**	**Managed Stray Cats (*n* = 210)**	**Unmanaged Stray Cats (*n* = 253)**
Sex	Female	110 (51.6%)	59 (28.1%)	183 (72.3%)
	Male	103 (48.5%)	64 (30.5%)	63 (24.9%)
	Unknown	0 (0%)	87 (41.4%)	7 (2.8%)
Coat length	Short	154 (72.3%)	172 (81.9%)	209 (82.6%)
	Medium	36 (16.9%)	25 (11.9%)	38 (15.0%)
	Long	23 (10.8%)	12 (5.7%)	4 (1.6%)
	Unknown	0 (0%)	1 (0.5%)	2 (0.8%)
Ear Tipped	Yes	N/A^*^	71 (33.8%)	0 (0%)
	No	N/A	121 (57.6%)	253 (100%)
	Unknown	N/A	18 (8.6%)	0 (0%)
Age	Juvenile	26 (12.2%)	17 (8.1%)	19 (7.5%)
	Adult	187 (87.8%)	193 (91.9%)	233 (2.19%)
	Unknown	0 (0%)	0 (0%)	1 (0.4%)

* *Note that owned cats aren't routinely ear tipped when being sterilized therefore, these are not reported as numbers*.

The 5-component visual health-related welfare assessment was found by the researchers to be easy to use. If the researcher had a clear view of the cat, the assessment was able to be performed in approximately 1–3 min per cat; some assessments took longer if the researcher had to wait for the cat to move (to assess lameness etc.). One of the challenges that the researchers faced was getting near enough to the stray cats to do an accurate assessment. In addition, often carers fed the cats at dusk or in the evening and the assessments could not be carried out under these conditions.

The majority of cats regardless of origin were in ideal body condition, good or excellent coat condition, and had no nose and eye discharge, ear crusting, or injuries (Table 2). No injuries were observed in 94.4% of CC (*n* = 201), 91.0% of MS (*n* = 191), and 92.5% of UMS (*n* = 234). The injuries that were observed and recorded were: missing eye (old injury), jaw injuries, lameness, scabs/lesions on nose, paralyzed tail, and wounds.

**Table 2:** Descriptive statistics on the 5-component visual health-related welfare assessment findings from 213 companion cats, 210 managed stray cats, and 253 unmanaged stray cats from Auckland, New Zealand.

		**Companion Cats (*n* = 213)**	**Managed Stray Cats (*n* = 210)**	**Unmanaged Stray Cats (*n* = 253)**
Body Condition	Emaciated	4 (1.9%)	1 (0.5%)	10 (4.0%)
	Thin	21 (9.9%)	34 (16.2%)	63 (24.9%)
	Ideal	161 (75.6%)	134 (63.8%)	163 (64.4%)
	Overweight	27 (12.7%)	35 (16.7%)	12 (4.7%)
	Over-condition	0 (0%)	0 (0%)	0 (0%)
	Unknown	0 (0%)	6 (2.9%)	5 (2.0%)
Coat Condition	Poor	2 (0.9%)	0 (0%)	21 (8.3%)
	Fair	11 (5.2%)	27 (12.9%)	70 (27.7%)
	Good	79 (37.1%)	140 (66.7%)	155 (61.3%)
	Excellent	120 (56.3%)	29 (13.8%)	5 (2.0%)
	Unknown	1 (0.5%)	14 (6.7%)	2 (0.8%)
Nose and Eye Discharge	None	203 (95.35)	179 (85.2%)	206 (81.4%)
	Mild	10 (4.7%)	16 (7.6%)	30 (11.9%)
	Moderate	0 (0%)	4 (1.9%)	14 (5.5%)
	Severe	0 (0%)	0 (0%)	3 (1.2%)
	Unknown	0 (0%)	11 (5.2%)	0 (0%)
Ear Crusting Score	None	213 (100%)	187 (89.0%)	223 (88.1%)
	Mild	0 (0%)	9 (4.3%)	23 (9.1%)
	Moderate	0 (0%)	1 (0.5%)	5 (2.0%)
	Severe	0 (0%)	0 (0%)	5 (2.0%)
	Unknown	0 (0%)	13 (6.2%)	0 (0%)
Injury Score	None	201 (94.4%)	191 (91.0%)	234 (92.5%)
	Mild	9 (4.2%)	9 (4.3%)	10 (4.0%)
	Moderate	3 (1.4%)	3 (1.4%)	6 (2.4%)
	Severe	0 (0%)	3 (1.4%)	1 (0.4%)
	Unknown	0 (0%)	4 (1.9%)	2 (0.8%)

## Discussion

This study was a preliminary investigation piloting a five-component objective visual health-related welfare assessment to assess the status of companion, managed stray, and unmanaged stray cats in Auckland, New Zealand. For all five indicators of welfare, the results suggest that the majority of companion, managed stray, and unmanaged stray cats in the study sample had reasonable welfare with ideal body condition score, good to excellent coat condition, no nose or eye discharge, no ear crusting, and no injuries. However, given the limitations with the sampling methods we cannot make accurate inferences about whether this represents the true welfare status of these cat populations in Auckland, New Zealand.

Although there is also evidence from other studies reporting good general health of stray or free-roaming cats (22, 23, 26–28), risk of infectious disease is a concern for the welfare of stray cats. However, the welfare assessments in this study generally found a relatively low incidence of visually obvious clinical signs that might be associated with infectious diseases. This is consistent with other studies which have found the baseline health status and prevalence of various infectious diseases in stray cats to be similar to that for companion cats. Nevertheless, the reported incidence of some infections varies (particularly Feline Immunodeficiency Virus and Feline Leukemia Virus) and may also be associated with the health status of the cat (13, 14, 20, 29–33). In future research, it would be ideal to collect more information about the disease prevalence in managed and unmanaged stray cat colonies. This would allow the exploration of the variation in disease prevalence between managed and unmanaged stray cat colonies and risk factors that may contribute to higher prevalence of disease in some colonies compared to others. This would also assist in developing evidence based best practice cat colony standards by helping to determine target values of disease prevalence and welfare indicators that managed colonies should be achieving to indicate that they are well-managed and that the cats have good welfare. Collection of blood from cats would allow the assessment of physiological parameters including routine biochemistry, disease prevalence, and indicators of stress such as cortisol. It would only be possible to do this without compromising cat welfare with cats who were sufficiently socialized to be handled for blood to be taken; some managed stray cats would likely fit this criterion but unmanaged stray cats would likely only be able to be sampled without welfare concerns if they were being trapped and sedated/anesthetized for other reasons, which would allow the collection of blood at the same time. For those cats who were not able to be handled for blood collection due to welfare concerns, non-invasive methods such as the quantification of fecal cortisol metabolites (FCMs) could possibly be used to perform some limited evaluation, particularly for unmanaged stray cats. Quantification of FCMs from fecal extracts using enzyme immunoassays has been validated and used in some wild felid species such as Bengal (Panthera tigris tigris) and Sumatran tigers (Panthera tigris sumatrae) (34–36). This has been recommended as a non-invasive method for evaluating the stress physiology of these wild cats and as an indicator of the health and welfare of these wild felids (34) and could be a useful and practical way to do the same for stray cats; this would be a valuable area for future research.

Welfare was assessed in this study through the assessment of relatively simplistic health-related indicators: the cats' body condition, coat condition, nose and eye discharge, ear crusting, and injuries. Although BCS is a relatively crude measure, previous studies have shown a link between body condition and overall animal health, behavior, and welfare (37–46). BCS is a relatively objective measurement, although it may be difficult to accurately assess through visual observation alone, particularly in long-haired cats. BCS can provide useful information associated with the health-related welfare of a cat because stress is often associated with a decrease in appetite and food intake in cats (47, 48). In addition, weight loss despite adequate food resources being available, is likely to be due to low food intake associated with stress and illness, and has been associated with the development of health problems such as upper respiratory tract infection in shelter cats (47). The relationship between stress, loss of weight, and resultant lower body condition score has implications for cat welfare that make BCS, which is a relatively easily assessed measure, a useful inclusion in welfare evaluations for cats. Nose and eye discharge score gives some indications about whether the cats are suffering from infectious disease that can affect their health and welfare; for example, feline upper respiratory tract infection (47, 49, 50). Ear crusting score can be an indicator of health problems that can negatively affect welfare, such as ear mite infection (*Otodectes cynotis*) or sun damage-related disease (for example, feline solar dermatosis or neoplasia such as squamous cell carcinoma) (51–53). Injury score can help to assess if the cats have suffered an accident or been involved in a physical altercation (54, 55).

A holistic assessment (including physical examination and health parameters, visual health-related welfare indicators, qualitative behavior assessment, and quality of life assessment) would be a valuable tool in determining the welfare of stray cats and informing cat management decisions. QoL relates to an individual's mental state, experiences, and the causes of their experiences (56–58). Recommendations for QoL assessment in veterinary practice and in veterinary research have been made (59); owners' perceptions of their cat's QoL have been reported (60) and owner reported care and behavior, and physical examination have been used to derive a QoL score for cats (61). Nevertheless, to the authors' knowledge, there have been no appropriate, objective, and validated QoL assessments developed specifically for cats, particularly stray cats. QoL scoring should, ideally, take into account the expressive quality of animal behavior and emotions by incorporating elements of qualitative behavior assessment (QBA). QBA relies on the assessor observing details of an animal's behavior and seeking to infer the animal's experience through the expressive nature of his/her demeanor (62–64). It would be important to involve animal behaviorists in the development of an assessment tool that included QBA and QoL scoring. In order for animal management to provide “acceptable” welfare, it is increasingly becoming recognized that positive welfare states must be promoted, as well as negative welfare states minimized (58, 65–69). It is important to consider what this means for stray cat management and, consequently, create a tool for assessing stray cat welfare that includes assessment of emotional as well as physical welfare measures.

There are certain welfare risks associated with the environment of free roaming cats (not just stray cats but also free roaming companion cats) that need to be considered but that are not likely to be adequately evaluated by individual animal welfare assessments. Disappearance or death, most often due to motor vehicle trauma, have been reported as common outcomes for stray cats (14, 23, 28). Accidental death is also generally the most common cause of mortality reported for companion cats with outdoor access, particularly younger cats (70–72). Cats who suffer significant injury or death, may simply disappear and so, cat welfare assessments are not necessarily a good way of evaluating environmental risks to welfare, particularly for stray cats (as their whereabouts may be less likely to be closely monitored compared to companion cats). Certain cat colony locations and situations are likely to be associated with a higher anthropogenic risk to cats (e.g., motor vehicle trauma and human cruelty), for example colonies that are situated in very built up areas and near busy roads (9, 15). These factors could affect the morbidity, mortality, and quality of life of the cats in a colony (9, 15). This highlights the need to assess the environmental risks to cat welfare at a specific site when selecting management options for a particular cat colony, as well as the well-being of the colony and its individual cats. Developing a tool for assessing environmental risk to cat welfare would assist in ethical cat management decision making.

Caution should be exercised when interpreting the results of this study due to the limitations and the preliminary nature of the 5-component visual health-related welfare assessment that needs validation through future research. A significant limitation to the study was that the cat observers were not blinded to the group from which the cats came. This introduces the potential for significant bias in the observations as the observers may unconsciously assign better welfare states to managed stray and companion cats. In future research intended to build on this preliminary study, observers collecting data about the cats should be blinded to the cats' group. While this would be difficult to achieve if the observers were physically present to see the cats (and hence able to infer from the environment and cats' behavior whether the cat was a companion, managed stray or unmanaged stray cat), this limitation could be overcome if photographs and videos were taken of cats and a secondary blind observer could rescore the cats' welfare measures. This could then be compared to the original scores from the non-blinded observer and would also allow formal evaluation of intra- and inter-rater variability, which was not performed when the current 5-component assessment system was developed. Another limitation of this study was the inability to account for the random effects when multiple cats from the same colony or household were sampled. It is suggested that researchers in the future use multistage random sampling to get a more accurate representation of colonies and cats.

Another limitation of this study that should be considered when planning any future research is that information on the date of sampling and the location of sampling was not recorded in the study database, which prevented robust analysis of the prevalence and risk factors for welfare. However, this was not the primary objective of this study. In addition, the data collection over the course of a full calendar year may have resulted in some seasonal variation. However, given the temperate Auckland climate, seasonal variation is likely to be minimal and most of the cats were fed directly or indirectly by humans, so the food source will have remained relatively constant.

Limitations related to the assessment tool itself included problems associated with assessing the cat demographics and health-related welfare measures visually and the lack of detailed descriptors for the different assessment measure categories. Coat condition is difficult to assess visually, and the perception of apparent differences may be influenced by types of coat and their coloring. Coat condition scoring should take these factors into account and also include tactile and close visual assessment of the coat condition. The ability to only visually assess cats made accurate identification of the sex of stray cats difficult. In the current study, only a small number of stray cats were ear-tipped, including some male cats without visible testicles and who were not ear-tipped. This made it impossible to conclusively determine visually if stray cats were sterilized. While it is possible that some sterilized male stray cats who were not ear-tipped were previously or currently owned (and therefore were not ear-tipped when they were sterilized), this finding could also suggest that there is a need to inform cat carers and veterinarians of the importance ear-tipping at the time of sterilization to ensure that stray cats are not unnecessarily trapped and sedated/anesthetized in order to carry out the same procedure. Regardless, it is still likely that the majority of stray cats were not sterilized. Previous research has suggested that sterilization is likely to reduce stress and improve welfare of stray cats (73–75). This is likely to be related, at least partly, to lower social and reproductive pressure and, consequently, less stress on the sterilized cats; as suggested by the reduced cortisol levels and aggression reported in sterilized stray female cats compared to entire stray female cats (73). In addition, roaming, fighting, and aggressive behaviors can be associated with higher risk of injury and infectious disease (72, 76, 77) and, as a result, poor welfare. Aggression, fighting, and roaming tend to decrease after sterilization (78, 79); this may contribute to the seemingly better welfare of sterilized stray cats compared to entire stray cats. It is suggested that future studies should develop a more detailed assessment tool that includes provision of specific descriptors for the different visual health-related welfare assessment categories, to ensure more consistency and accuracy, and that formal statistical testing of inter-and intra-rater reliability is conducted.

For future research, it is suggested that ethics approval be sought to permit physical examination of the cats where possible; this would allow for more accurate assessment of both demographic variables and health/welfare indicators. However, this would need to be balanced with the need to maintain acceptable welfare for cats who are unused to being handled. Unmanaged stray cats are unlikely to be able to be sufficiently socialized to allow this, and so it would only be possible to perform physical examination of these cats if they were being trapped and sedated/anesthetized for other reasons which would allow for examination at the same time. The unmanaged stray cats in this study were all in traps when they were assessed but full physical examination was not performed due to the more limited scope of the study and ethics approval.

The negative welfare of stray cats has been raised as an objection to the use of TNR programmes to manage their populations (7, 11). However, the results from this preliminary study suggest that the welfare of the stray cats studied in Auckland was reasonable, particularly the managed stray cats. There is also evidence from other studies suggesting that generally human care provided to stray cats has positive effects on the cats' health and welfare (14, 15, 23, 73, 75, 80, 81). More evidence of the benefits of human assistance to stray cat health and welfare are reported in the Newburyport, Massachusetts trap-neuter-return case study (22). In the Newburyport programme, which also included the feeding of and monitoring/caring for cats, all of the stray cats in the targeted area were sterilized over the years of the programme and, over time, the general health of the cats improved (22). The development of best practice guidelines for the management of stray cat colonies and TNR programs could be one way to encourage care that would provide good welfare for stray cats. In addition, a stray cat colony register and a requirement for stray cat carers to register and abide by the best practice guidelines (4) could further improve the welfare and health of stray cats. The evidence seems to suggest that, where stray cats are allowed to continue to reside in an area, it would be of benefit to encourage management of the colony (so that the cats are sterilized and consistently fed, monitored, and cared for).

## Conclusion

Even though there were considerable limitations with the sampling methods and assessment tool for this study, the findings suggest that stray cats–particularly managed stray cats–can have reasonable welfare that is potentially comparable to companion cats. Therefore, maintaining stray cats in managed colonies where cats are sterilized and consistently fed, monitored, and cared for may be a way to promote good welfare and a positive quality of life for stray cats where non-lethal management is possible and appropriate. However, such a system would need to be carefully managed and colonies judiciously selected.

Information on stray cat welfare is largely unreported but the welfare of stray cats in the field has very important implications for policy development. The ability to generalize the findings from this study is limited by the small sample size and the limitations of the assessment tool and data collected. Nonetheless, despite this research being very preliminary in nature, it provides a starting point for further research that is urgently needed in this area. It would be of benefit if future studies could develop a validated welfare assessment for cats including both visual health-related welfare indicators and QoL assessment; this would likely necessitate comparing visual health-related welfare indicators and QoL assessment scores with physical welfare measurements. The discussion of the limitations and suggestions for prospective research should assist researchers to improve the design of future investigations in this field, including collecting more variables about the managed and unmanaged colonies to help accurately assess the policy implications of the welfare of stray cats and how best to manage them.

## Ethics Statement

Although animals were the subject of this study, animal ethics approval was not required as this was a purely observational study. In NZ, animal ethics approval is only required for animals that are being manipulated for the purpose of research, testing, and teaching, which was not the case with the cats in this study. Approval from the Unitec Human Ethics Committee was obtained for gaining informed consent from owners to participate in collecting data about their cats.

## Author Contributions

AD and JW oversaw the design and implementation of the study and assisted with data collection. SZ and MG analyzed the data. SZ, JW, MG, and AD wrote the paper and reviewed the manuscript.

### Conflict of Interest Statement

JW is currently an employee of the New Zealand Companion Animal Council which is axillary to the New Zealand Companion Animal Trust. JW was not employed by the New Zealand Companion Animal Council when this research was conceived, funded, and conducted, and the funding body had no role in any aspects of the study design, data collection, analysis, interpretation of the data, the writing of the manuscript, or in the decision to publish the results. AD is the current Chair of the New Zealand Companion Animal Trust. AD was not in this role or employed by the New Zealand Companion Animal Council when this research was conceived, funded, and conducted, and the funding body had no role in any aspects of the study design, data collection, analysis, interpretation of the data, the writing of the manuscript, or in the decision to publish the results. The remaining authors declare that the research was conducted in the absence of any commercial or financial relationships that could be construed as a potential conflict of interest.
